# Early Invasive Cancer and Partial Intraoperative Electron Radiation Therapy of the Breast: Experience of the Jules Bordet Institute

**DOI:** 10.1155/2014/627352

**Published:** 2014-06-09

**Authors:** C. Philippson, S. Simon, C. Vandekerkhove, D. Hertens, I. Veys, D. Noterman, F. De Neubourg, D. Larsimont, P. Bourgeois, P. Van Houtte, J. M. Nogaret

**Affiliations:** ^1^Department of Radiation Oncology, Jules Bordet Institute, 1000 Brussels, Belgium; ^2^Department of Radiophysics, Jules Bordet Institute, 1000 Brussels, Belgium; ^3^Department of Surgery, Jules Bordet Institute, 1000 Brussels, Belgium; ^4^Department of Pathology, Jules Bordet Institute, 1000 Brussels, Belgium; ^5^Department of Nuclear Medicine, Jules Bordet Institute, 1000 Brussels, Belgium

## Abstract

*Objectives*. The aim of this prospective phase II study is to evaluate the treatment of early-stage breast cancer (T1 N0) with intraoperative electron radiation therapy (IOERT) in terms of local control, early complications, and cosmesis. *Patients and Methods*. From February 2010 to February 2012, 200 patients underwent partial IOERT of the breast. Inclusion criteria were unifocal invasive ductal carcinoma, age ≥40 years, histological tumour size ≤20 mm, and no lymph node involvement. A 21 Gy dose was prescribed over the 90% isodose line in the tumour bed. Median follow-up is 23.3 months (7–37). *Results*. Acute toxicity was not frequent (Grade 1: 4.5%, Grade 2: 1%). The cosmetic result was considered to be very good or good in 92.5%. One ipsi lateral out-quadrant recurrence at 18 months was observed. The crude and actuarial local recurrence rates after median follow-up were 0.5% and 0.9%, respectively. *Conclusion*. The preoperative diagnostic work-up must be comprehensive and the selection process must be rigorous for this therapeutic approach reserved for small ductal unifocal cancers. After a 23.3-month median follow-up time, the clinical results of IOERT for selected patients are encouraging for the locoregional recurrence and the toxicity rates. The satisfaction of our patients in terms of quality of life was extremely high.

## 1. Introduction

Breast cancer is the most common cancer affecting women as it accounts for over one-third of all cancers reported in this population. Although the cure rate is high, the sheer number of new cases detected every year means that the mortality rate associated with this cancer remains high. Advances in diagnostic methods and widespread screening campaigns have made that these tumours are now being detected at an earlier stage and are more frequently eligible for conservative treatment. However, numerous studies [1–3] have demonstrated the need for irradiation of the entire mammary gland combined with a boost delivered to the tumour bed after completing surgical treatment. The local recurrence rate at 10 years with this conventional approach is estimated to be between 5 and 10%. In 85% of cases, the recurrence is located in the original tumour bed [4]; in the remaining 15%, it is detected elsewhere in the breast and is probably a new tumour [5–7].

It should be pointed out that breast cancer cells should be more sensitive to short bursts of intense radiation than to small doses fractionated over several weeks. In order to try to enhance efficacy through increasingly targeted treatments, numerous studies have been performed exploring the outcomes of higher radiation doses delivered over shorter treatment periods [8–10]. Several partial radiation techniques, targeting the at-risk portion of the breast only, have also emerged. The ASTRO [11] and ESTRO [12] have already published guidelines to help practitioners identify low-risk women who can be treated with these techniques outside clinical trials.

Intraoperative electron radiation therapy is a particularly suitable option since it permits delivery of the whole radiation dose directly to the target zone liable to contain residual cancer cells, clearly visible during surgery, from which all the healthy structures have been carefully separated. Another advantage is efficiency: in 2 minutes a radiation dose with the same tumoricide potential as 6 weeks of conventional treatment can be given with the additional advantages of enhanced quality of life, and no irradiation of the skin, lungs, heart, and ribs. Oncoplastic surgery can be performed if needed and if systemic treatment is indicated, it will not be delayed.

## 2. Patients and Methods

Between February 2010 and February 2012, 200 patients (median age: 61 y, range: 40–85 y) underwent partial intraoperative electron radiation therapy of the breast in our Institute (Table 1). Inclusion criteria were unifocal (demonstrated by preoperative MRI) invasive ductal carcinoma, age ≥40 years, histological tumour size ≤20 mm, and no lymph node involvement. All histologic grades (I-II-III) and all hormonal receptor types (RH+, RH−) were accepted. Tubular, colloidal, mucinous, and medullary types were also included. Invasive lobular carcinoma was excluded as were patients with lymphovascular involvement and extensive intraductal disease.

### 2.1. Preoperative Work-Up

The histological diagnosis was based on microbiopsy. Conventional imaging as well as MRI of the breast was used to rule out multifocal disease. All patients underwent a metastatic work-up including a chest X-ray, ultrasound of the liver, bone radionuclide scan, and a blood test.

### 2.2. Surgical Technique

The surgical procedure began with removal of the sentinel lymph node(s) (SLN), which were identified with a gamma probe. A lumpectomy was then performed, via an elliptical skin incision made directly over the tumour. This allows introducing the radiation applicator through the incision and offers optimal control of the anterior surgical margin. It also makes it possible to mobilize the target glandular tissues surrounding the lumpectomy bed for radiation. The tumour was removed in one piece with a 1 to 2 cm safety margin extending posteriorly, where possible, to the aponeurosis of the pectoralis major muscle. The surgical specimens inclusively sentinel node were sent to the pathology laboratory for intraoperative analysis of tumour size, surgical resection margins, and SLN malignancy. If the histological criteria were met (pT1, safe margins ≥1 mm, pN0), the target tissue was dissected from the underlying pectoralis major aponeurosis in order to be able to put a protective shield in place. The shield consisted of 3 mm of lead (deep part of the breast) and 4 mm of aluminium (anterior part of the gland) so that all the electrons were intercepted by the lead and the electrons back-scattered by the lead were blocked by the aluminium. The total thickness of the shield is equivalent to more than 45 mm of water for electron slowing down. This completely stops 9 MeV electrons, independently of the treated thickness. For 12 MeV electrons, which are only used if the thickness to be treated is bigger than 29 mm, the combined equivalent thickness of treated tissues and shield is large enough to completely stop the electrons. The gland was then dissected of the subcutaneous fat and skin and as much of the breast tissue potentially containing residual microscopic cancerous foci as possible brought into the tumour bed: these tissues were sutured over the shield [13]. The shield generally used was 15 to 20 mm larger in diameter than the applicator whose diameter was already 40 mm larger than the tumour itself (size of the breast permitting), in order to create a 20 mm safety margin around the tumour bed. Although the sentinel lymph node (SLN) operating pathology was always negative in frozen section and immunohistochemical analysis [14], the final pathology showed positive SLN in 6.9% of the patients (pN1mic: 3.9%, pN1a: 3%) (Table 1). The immunohistochemical analysis is an asset to keep the false negative rate low but it has not been possible to reach a lower rate in this first study. For the pN1a patients, we performed complete axillary node dissection (CAND) except for one 80 y luminal A patient for whom 4 other lymph nodes were already removed during the SLN procedure. We did not perform CAND for the pN1mic patients. The patients presenting with micro- or macroscopic spread to the sentinel node were not treated with external radiation therapy.

### 2.3. Radiation Therapy

All patients were treated with electrons generated by an IntraOp (Mobetron) dedicated mobile accelerator. The diameter of the cylindrical applicators available varied from 3 to 10 cm, in 0.5 cm increments. The applicator most frequently used had a diameter of 50 mm (in 36.8% of the cases) (range: 35–65 mm). As mentioned before, our IOERT PTV policy was adapted to the tumour size. As a general rule, the field diameter used was at least 40 mm bigger than the pathological tumour diameter. The dose delivered was 21 Gy, prescribed over the 90% isodose line as described in the dose escalation studies conducted by the European Institute of Oncology in Milan [15]. The 90% isodose line diameter at dmax is slightly smaller than the nominal field diameter (from 6 to 9 mm smaller, depending on field size and energy). Taking that into account, we have around the tumour bed a risk-adapted treated volume whose diameter is at least 36 mm for pT1a tumours, 41 mm for pT1b, and 46 mm for pT1c tumours. The applicator extremity was either flat or bevelled (15° to 30°). Electron energies of 4, 6, 9, and 12 MeV were available. The 9 MeV was the energy the most frequently selected (in 48% of the cases) (range 6–12 MeV). A 5 or 10 mm bolus was used either to increase the entrance dose to at least 90% or to decrease the total electron range in the patient. Energy was determined in function of the maximum thickness of the target tissue, in order to have the 90% isodose depth greater than the maximum target thickness. The maximum target thickness was simply measured by introducing a needle into the tissue down to the protective shield placed over the muscle. Beam calibration was performed on the treatment day for quality control purposes. The position of the protective shield was controlled intraoperatively by inserting a needle into the gland at a tangent to the applicator and ensuring that it “hit” the shield. During radiation, a digital radiograph was positioned on the Mobetron beam-stopper to maintain an exact record of the shield's position relative to the applicator. Dosimeters were positioned on the contralateral breast, the thyroid gland, and ovaries to determine, under* in vivo* conditions, the exact dose received by these sensitive structures at a distance from the beam (Table 2).

### 2.4. Systemic Treatments

These were determined by the molecular subtype of the tumours and the comorbidities presented by the patients. Most of the tumours (181 or 88.7%) were molecular subtype luminal A or luminal B (73.5% and 15.2%, resp.). In thirteen cases (6.4%) the her2/neu oncogene was amplified and 9 (4.4%) were of the triple negative subtype (Table 1).

Genomic analysis was recommended for patients with intermediate grade histological disease [16]. Hormone therapy alone was prescribed for 163 patients (81.5%) and chemotherapy alone for 9 patients (4.5%). Twenty-four patients (12%) received both treatments. Targeted therapy was prescribed for all 9 patients with amplified her2/neu oncogene. Three patients (1.5%) were not prescribed any adjuvant treatment in the view of their low risk and major comorbidities. One patient (0.5%) did not receive any adjuvant treatment because her hormonal receptor status was unknown (3 mm tumour removed during microbiopsy).

### 2.5. Toxicity

Acute toxicity (perioperative and up to 30 days) was assessed as per NCI Common Terminology Criteria for Adverse Events version 3.0 [17] and was acceptable (Grade 1: 4.4%, Grade 2: 1%) (Table 3).

Grade 1 late toxicity according to the LENT SOMA scale [18] was reported in 13.7% of treatments and Grade 2 in 3.4% (Table 4).

### 2.6. Cosmetic Results

Cosmetic outcome was assessed both by the doctor and the patient at last 7 months after treatment, based on the cosmetic scoring system proposed by Beal et al. [19]. The cosmetic criteria were breast symmetry, breast oedema, discoloration at site, dimpling/local contour change, and scar prominence. The cosmetic result was considered to be very good (all Grade 0) in 124 patients (62%), good (any Grade 1) in 61 patients (30.5%), fair (any Grade 2) in 11 patients, and poor (any Grade 3) in 4 patients (2%) (Table 5). Poor results could be corrected by plastic surgery if the patient so wished.

## 3. Results

Two patients died due to another cancer. Among the 198 patients alive, 2 presented with another cancer, none of them developed metastasis due to breast cancer.

One local out-quadrant recurrence was observed; no regional recurrence was detected.

The crude and actuarial local recurrence rates after median follow-up were 0.5% and 0.9%, respectively.

The locoregional recurrence rate was 0.5%.

The actuarial rates after median observation time for DFS (disease free survival), OS (overall survival), and DSS (disease specific survival) were 97.6%, 98.9%, and 98.9%, respectively.

Acute toxicity was reported in 5.4%. Late toxicity was observed in 17.1%.

The cosmetic outcome was considered to be very good or good in 92.5%.

## 4. Discussion

We know that the risk of recurrence is the highest in the tumour bed [4]. We also know that the percentage of residual tumour cells decreases with increasing distance to the tumour bed. Moreover, the risk of local recurrence and lymph node involvement is higher in patients with extensive intraductal disease [20, 21]. It therefore follows that local control of the malignant disease and, secondarily, increasing survival rates are the primary objectives of any “innovative” technique. It is also important to reduce the risks of radiation-induced complications such as skin fibrosis, a poor cosmetic result, cardiac toxicity, pneumonia, and secondary cancers. At the present time for local dose augmentation to the tumour bed, delivered intra- or perioperatively, a numerous treatment techniques are available which show great differences in their respective dose homogeneities [22].

The undeniable advantage of these nonintraoperative techniques is that they are implemented in full knowledge of the definitive anatomopathology results. The major inconveniences are the significant radiation dose delivered to healthy organs and the potential presence of hot spots.

In view of the numerous existing techniques and as a result of the growing interest worldwide for this approach, the American (ASTRO, American Society for Radiation Oncology) and European learned societies (GEC-ESTRO, Groupe Européen de Curiethérapie—European Society for Therapeutic Radiology and Oncology) have issued guidelines defining low-risk patient groups (age over 50 or 60 years old, size ≤20 or 30 mm, healthy sentinel node, ductal type, and unicentric tumour) which could be treated outside clinical trials.

The Milan team recently presented their results for the randomised ELIOT trial [23] in which they include 1305 patients (654 to external radiotherapy and 651 to intraoperative radiotherapy). Their inclusion criteria were age 48–75 years, early breast cancer, tumour diameter up to 25 mm, and suitable for breast conserving therapy. The 5-year event rate for ipsilateral breast tumour recurrences (IBTR) was 4.4% in the intraoperative radiotherapy group and 0.4% in the external radiotherapy group. The authors stated that the significant difference in local recurrence is probably attributable to the very low IBRT rate they achieved in their external radiotherapy arm. They identified subgroups with negative prognostic factors for IBTR: tumour diameter >20 mm, four or more positive nodes, Grade 3 tumour, and triple negative tumour. Each of these factors is associated with a 5-year IBTR >10% but the authors stated that these factors require further validation. For example, some consider that the local recurrence rate for patients with triple negative disease does not appear to be higher than for the other subtypes [24] although recurrence is observed at an earlier date (within two years). This is why we did not exclude this patient profile from our study. For the 452 patients (69.4%) in the ELIOT trial, who had none of these factors, the 5-year IBTR in the IOERT arm was only 1.5%. In our series, regarding these negative prognostic factors, 26 patients (13%) presented one of them (24 Grade 3 alone and 2 triple negative alone) and 7 patients (3.5%) had 2 of them (both Grade 3 and triple negative). None of them recurred but our follow-up is too short and the number of patients too limited to conclude. In order to decrease the IBTR, the Milan team suggested considering giving additional external whole breast irradiation if a negative prognostic factor was found after final histological examination.

Our IBTR at 23 months is comparable with the ELIOT study (0.9% versus 1%) or with the TARGIT Trial [25] (0.72% at 29 months). The same conclusion applies for the overall survival (98.9% versus 99% for ELIOT or versus 99.1% for TARGIT). Our annual IBTR rate of 0.5% is in the range (<1%) of what is commonly accepted according to European guidelines after breast conserving therapy [26].

Concerning our case of local recurrence, it was a 63-year-old patient presented initially with an upper internal quadrant primary invasive ductal carcinoma at 10 mm from the nipple-areolar complex, measuring 8 mm, SLN negative, Luminal A subtype (grade I, RH+, Ki 67 less than 10%, HER/neu−) for which she had been prescribed hormone therapy (tamoxifen). The disease recurred in the upper external quadrant, close to the nipple-areolar complex. She underwent mastectomy and breast reconstruction (DIEP-flap) followed by second line hormone therapy with an aromatase inhibitor. The pathologic examination found a 6 mm invasive ductal carcinoma, molecular subtype Luminal A (grade II, Ki 67 10%, positive hormonal receptors (RO 7/8, RP 5/8), HER/neu−) with 5 negative lymph nodes. It should be noted that it turned out to be extremely superficial, subcutaneous, and diagnosed sonographically only. It could in fact have been progression of a microfocus which had not been detected during the initial work-up rather than a genuine recurrence. Histological analysis shows that this recurrence was located at distance from the site of the earlier lumpectomy. It should also be noted that this particular patient had an important family history (mother and daughter). Our case of recurrence more closely resembles progression of a microfocus since it appeared 18 months after the diagnosis in a patient who, other than her family history, did not present any poor prognosis factors. It is also possible that too much subcutaneous tissue was left in place.

## 5. Conclusion

PBI according to the ELIOT-concept which was performed in our Institution is a feasible technique with good treatment tolerance resulting in a sufficient cosmetic outcome. However, our median follow-up is too short to reach any conclusions in terms of recurrence and survival.

## Figures and Tables

**Table 1:** Characteristics of our patients.

Parameters	Characteristics	*N* = 200 patients
Mean follow-up (months)		23 (7–37)

Age (years)	40–49	18 (9%)
	50–60	75 (37.5%)
	≥60	107 (53.5%)

Side	Left	104 (52%)
	Right	92 (46%)
	Bilateral	4 (2%)

Localisation	UOQ	72 (35.3%)
	UIQ	26 (12.8%)
	LEQ	18 (8.8%)
	LIQ	10 (4.9%)
	Upp jct	29 (14.2%)
	Ext jct	9 (4.4%)
	Int jct	8 (3.9%)
	Inf jct	25 (12.3%)
	Central	7 (3.4%)

Histology	Ductal	192 (94.1%)
	Mucinous	4 (2%)
	Tubular	1 (0.5%)
	Mixed	1 (0.5%)
	Lobular	6 (2.9%)

Size AP (mm)	≤5 pT1a	6 (3%)
	6–10 pT1b	75 (36.8%)
	11–20 pT1c	118 (57.8%)
	≥21 pT2	5 (2.4%)

Margins	Negative	202
	Positive (invasive)	1
	Positive (in situ)	1
	Negative after reexcision	204 (100%)
	Vascular emb	10 (4.9%)

Grade	I	88 (43.1%)
	II	85 (41.7%)
	III	31 (15.2%)

Molecular subtype	Luminal A	150 (73.5%)
	Luminal B	31 (15.2%)
	HER2/neu+	13 (6.4%)
	Triple negative	9 (4.4%)
	Not useful	1 (0.5%)

SLN status	pN0 negative	190 (93.1%)
	pN1mic positive	8 (3.9%)
	pN1a positive	6 (3%)

Number of N+	0	190 (93.1%)
	1	14 (6.9%)

Cancer related events	Local recurrence	1 (0.49%)
	Regional recurrence	0
	Distance recurrence	0
	Contralateral cancer	0
	Other cancers	4 (2%)
	Total	**5 (2.5%)**
	Death from breast cancer	0
	Death from other cancers	2 (1%)
	Death from other causes	0
	Total	**2 (1%)**

**Table 2:** Doses [cGy] for 196 PBI (bilateral not included).

	Thyroid	Contralateral breast	Ovaries
Mean	0.89	0.39	0.13
St. dev.	0.64	0.22	0.10
Min.	0.08	0.02	0.04
Max.	3.20	1.97	1.19

**Table 3:** Acute toxicities according to NCI Common Terminology Criteria for Adverse Events version 3.0.

	Grade 1	Grade 2	Grades 3-4-5
Infection	3 (1.5%)	0	0
Haematoma	3 (1.5%)	0	0
Delayed cicatrisation	2 (1%)	2 (1%)	0
Local inflammation	1 (0.5%)	0	0

**Table 4:** Late toxicities according to LENT SOMA scoring scale.

	Grade 0	Grade 1	Grade 2	Grades 3-4
Fibrosis	0	17 (8.3%)	7 (3.4%)	0
Atrophy	0	11 (5.4%)	0	0

**Table 5:** Cosmetic results.

	At latest follow-up examination (min 7 months)
Grade 0 (no effects)	124 (62%)
Grade 1	
Minimal asymmetry	61 (30.5%)
Minimal edema	—
Mild* discoloration at site	—
Mild* dimpling/local contour change	—
Mild* scar prominence	—
Grade 2	
Asymmetry (≤1/3 of the gland)	11 (5.5%)
Edema (≤1/2 of the gland)	—
Discoloration at site (≤1/3 of the gland)	—
Dimpling/local contour change (≤1/3 of the gland)	—
Scar prominence (moderate, thickened, or raised)	—
Grade 3	
Asymmetry (>1/3 of the gland)	4 (2%)
Edema (>1/2 of the gland)	—
Discoloration at site (>1/3 of the gland)	—
Dimpling/local contour change (>1/3 of the gland)	—
Scar prominence (severe)	—

*Notable only with close inspection.
